# Identification and Comparative Quantification of Bio-Active Phthalides in Essential Oils from Si-Wu-Tang, Fo-Shou-San, Radix Angelica and Rhizoma Chuanxiong

**DOI:** 10.3390/molecules15010341

**Published:** 2010-01-15

**Authors:** Yuping Tang, Min Zhu, Sheng Yu, Yongqing Hua, Jin-Ao Duan, Shulan Su, Xu Zhang, Yin Lu, Anwei Ding

**Affiliations:** Jiangsu Key Laboratory for TCM Formulae Research, Nanjing University of Chinese Medicine, Nanjing 210046, China

**Keywords:** Si-Wu-Tang, Fo-Shou-San, Radix Angelica, Rhizoma Chuanxiong, phthalide, gas chromatography–mass spectrometry

## Abstract

Phthalides are important bio-active constituents in Si-Wu-Tang and Fo-Shou-San, two commonly used Traditional Chinese Medicine (TCM) combined prescriptions mainly derived from Radix Angelica and Rhizoma Chuanxiong. In this paper, the contents of eight phthalides, including *Z*-ligustilide, *E*-ligustilide, *Z*-butylenephthalide, *E*-butylene-phthalide, 3-butylphthalide, neocnidilide and senkyunolide A were determined or estimated by gas chromatography-mass spectrometry (GC-MS). The results showed GC-MS was a simple, rapid, and high sensitive method for analyzing phthalides in Si-Wu-Tang, Fo-Shou-San, Radix Angelica and Rhizoma Chuanxiong, and the extractable contents of each phthalides including *Z*-ligustilide, *E*-ligustilide, *Z*-butylenephthalide, *etc*. varied after Radix Angelica, Rhizoma Chuanxiong were combined into a formulation, such as Si-Wu-Tang and Fo-Shou-San. Furthermore, inhibition activity of essential oils from Si-Wu-Tang, Fo-Shou-San, Radix Angelica and Rhizoma Chuanxiong on uterine contraction was tested in an *in vitro* assay, and the results showed that the activity of the essential oil is higher as the content of the phthalides increase, which demonstrated that phthalides are possibly main active components inhibiting mice uterine contraction *in vitro*. All of the results suggested that comparative analysis of chemical components and pharmacological activities of each herb and formula is possibly helpful to elucidate the active components in traditional Chinese medicine, and to reveal the compatibility mechanism of TCM formulae.

## 1. Introduction

In clinical application, most Traditional Chinese Medicines (TCMs) are prescribed in combination to obtain the synergistic effects or to diminish the possible adverse reactions [1,2]. This medical approach has played an important role in the prevention and treatment of diseases. The dried radix of *Angelica sinensis* (Oliv.) Diels and rhizomes of *Ligusticum chuanxiong* Hort have been widely used in TCM to treat some pathological conditions such as atherosclerosis and hypertension [3,4,5]. On the basis of Radix Angelica (RA) and Rhizoma Chuanxiong (RL), many formulae are composed under the guidance of traditional Chinese medical philosophy, such as Si-Wu-Tang (RA, RL, Radix Rehmanniae, and Radix Paeonia at the ratio of 1:1:1:1) and Fo-Shou-San (RA and RL at the ratio of 1:1), which have been used as the hematic and blood-activating medicine, and to treat emmeniopathy for hundreds of years and widely adopted for clinical use in China and Japan. Because Si-Wu-Tang (SWT), Fo-Shou-San (FSS), RA and RL have diverse therapeutic effects, their constituents [6,7,8,9,10,11,12,13,14] have been widely studied. There are many kinds of bio-active constituents in SWT, FSS, RA and RL, including phthalides (e.g., *Z*-ligustilide, *Z*-butylenephthalide, and senkyunolide A), phenolic constituents (e.g., ferulic acid, coniferyl ferulate), *etc*. Phthalides, a characteristic type of compounds, were thought to be responsible for the majority of the types of bioactivity reported [15]. For example, *Z*-lingustilide was most commonly reported bioactive phthalide in different assays that included activity as an antifungal, antibacterial, antiasthmatic, anti-brine shrimp, anti-inflammatory, antioxidant and insecticidal. Moreover, it was suggested to have multidrug-resistance modulation, phytotoxic, smooth muscle relaxant, and vasodilation activities. Other phthalides such as butylidenephthalide and senkyunolide A also showed similar bioactivity [16]. When RA and RL were combined into a formula with other medicinal herbs, there will be some possible change in the content of extractive phthalides [17,18].

The present study mainly focuses on identification and comparative quantification of bio-active phthalides in essential oils obtained from SWT, FSS, RA and RL by hydrodistillation, followed by gas chromatographic-mass spectrometric analysis to investigate the variation in the extractable contents of the main phthalides, including *Z*-ligustilide, *E*-ligustilide, *Z*-butylenephthalide, *E*-butylenephthalide, 3-butyl-phthalide, compound 6, neocnidilide and senkyunolide A after RA, RL were combined into a formulation. Moreover, the inhibitory activity of the essential oils from SWT, FSS, RA and RL on uterine contraction *in vitro* was compared, and its correlation with phthalide content in different essential oils was also analyzed.

## 2. Results and Discussion

### 2.1. Identification of chemical components in the essential oils

**Figure 1:**
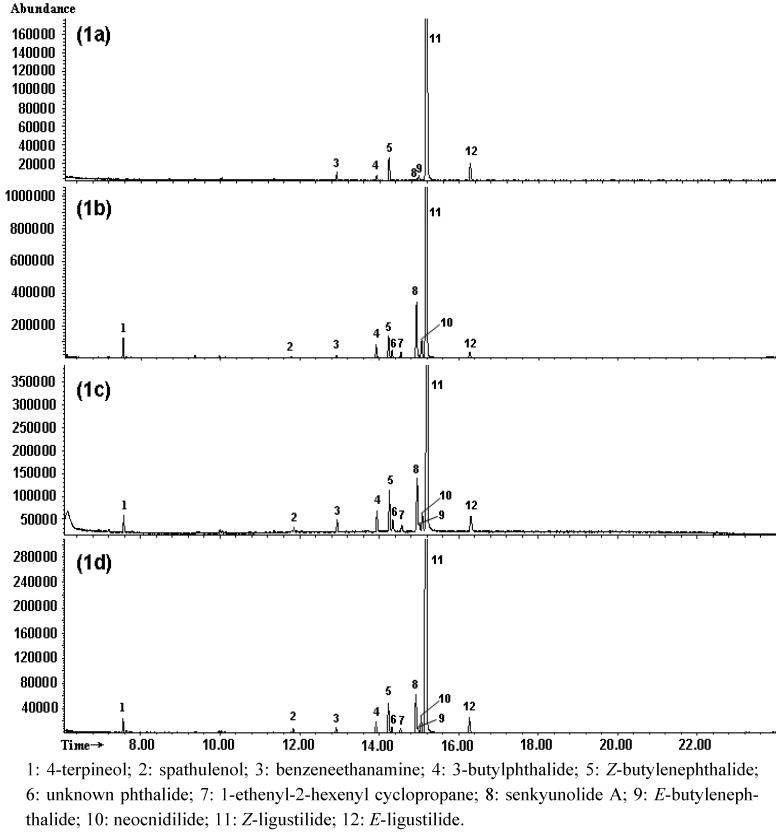
GC-MS total ion chromatograms for essential oils from RA (1a), RL (1b), FSS (1c), and SWT (1d).

**Figure 2:**
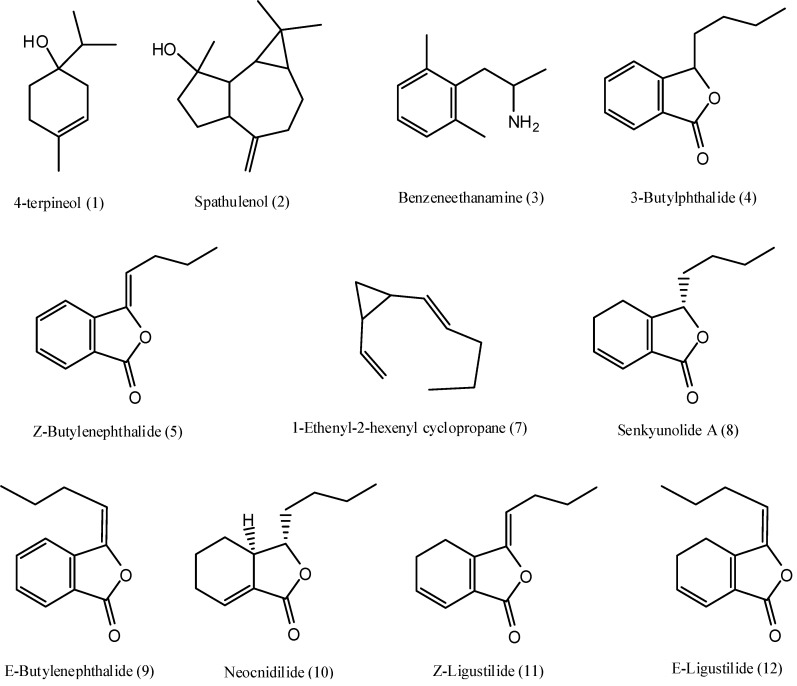
The structures of twelve compounds identified in essential oils from SWT, FSS, RA and RL.

The total ion chromatograms (TIC) of essential oils from SWT, FSS, RA and RL, are shown in Figure 1. All the main components were separated completely in 20 min, and twelve of them (including a unknown phthalide corresponding to peak 6) were identified on the basis of comparison of their mass spectra with NIST05 database through MSD ChemStation D.05.01, or with mass spectra of standard compounds (3-butylphthalide, *Z*-butylenephthalide, senkyunolide A, and *Z*-ligustilide) and data reported in the literature. Peaks 1-12 were identified as 4-terpineol, spathulenol, benzeneethanamine, 3-butylphthalide [19], *Z*-butylenephthalide [19], an unknown phthalide, 1-ethenyl-2-hexenyl cyclopropane, senkyunolide A [19,20], *E*-butylenephthalide, neocnidilide [19], *Z*-ligustilide [19,20], and *E*-ligustilide [19], respectively. The results are listed in Table 1, and the structures of the identified compounds are shown in Figure 2.

**Table 1:** MS data of 12 compounds identified from SWT, FSS, RA and RL.

Peak No.	Compound	Rt (min)	MS data^a^
**1**	4-Terpineol	7.56	154 (M^+^, 20), 136 (12), 111 (60), 93 (53), 91 (20), 86 (24), 69 (25), 67 (23), 55 (30), 44 (30), 43 (54)
**2**	Spathulenol	11.85	220 (M^+^, 26), 206 (22), 205 (100), 191 (8), 145 (9), 105 (11), 81 (10), 57 (16), 44 (33), 43 (17)
**3**	Benzeneethanamine	12.93	205 (M^+^, 33), 159 (25), 149 (20), 133 (17), 131 (26), 119 (22), 105 (34), 91 (38), 44 (100), 43 (71)
**4**	3-Butylphthalide	13.93	190 (M^+^, 3), 134 (11), 133 (100), 105 (27), 77 (12), 76 (4), 51 (5), 44 (10), 43 (4)
**5**	*Z*-Butylenephthalide	14.23	188 (M^+^, 19), 173 (2), 160 (12), 159 (100), 146 (31), 131 (23), 115 (7), 104 (15), 103 (22), 77 (19)
**6**	Unknown phthalide	14.32	192 (M^+^, 84), 163 (13), 159 (17), 150(29), 149 (43), 133 (19), 122 (20), 108 (100), 107 (59), 76 (62), 44 (81)
**7**	1-Ethenyl-2-hexenyl cyclopropane	14.55	150 (M^+^, 15), 107 (13), 94 (41), 93 (55), 91 (27), 80 (53), 79 (100), 77 (29), 44 (50), 43 (25), 41 (17)
**8**	Senkyunolide A	14.93	192 (M^+^, 20), 163 (2), 133 (19), 108 (10), 107 (100), 105 (10), 85 (8), 79 (25), 77 (30)
**9**	*E*-Butylenephthalide	14.99	188 (M^+^, 19), 160 (16), 159 (100), 146 (40), 133 (15), 131 (24), 107 (17), 103 (31), 77 (25), 44 (49)
**10**	Neocnidilide	15.06	194 (M^+^, 2), 137 (4), 109 (14), 108 (100), 91 (3), 81 (8), 80 (20), 79 (31), 77 (9), 44 (10), 41 (6)
**11**	*Z*-Ligustilide	15.18	190 (M^+^, 63), 161 (100), 148 (86), 147 (15), 134 (18), 120 (13), 115 (11), 106 (41), 77 (34), 55 (52)
**12**	*E*-Ligustilide	16.27	190 (M^+^, 71), 161 (100), 159 (28), 148 (83), 147 (17), 133 (20), 106 (46), 105 (72), 77 (44), 55 (64)

^a^ *m/z*, relative intensity is shown in parenthesis, and ion of relative intensity 100 was used for the quantification.

### 2.2. Quantification of eight phthalides

The selected ion monitoring (SIM) method was used for the quantification of eight phthalides in SWT, FSS, RA and RL. The fragment ions with *m/z* 133, 159, 108, 107, 159, 108, 161 and 161 were used for 3-butylphthalide, *Z*-butylenephthalide, compound 6, senkyunolide A, *E*-butylenephthalide, neocnidilide, *Z*-ligustilide and *E*-ligustilide, respectively. As phthalide analogues, the content of 3-butyl-phthalide, *Z*-butylenephthalide, compound 6, senkyunolide A, *E*-butylenephthalide, neocnidilide and *E*-ligustilide in SWT, FSS, RA and RL, were estimated by using calibration curve of *Z*-ligustilide, which is the major phthalide derivative found in these TCMs. Though the approach could lead to over or underestimation of the other phthalides if they do not ionize in the ion source of MS to the same degree as *Z*-ligustilide, the quantified data of these phthalides does not affect the conclusions of this study because those were determined under the same GC-MS conditions, and were used just for comparison purposes [21].

The calibration curve, which obtained from the selected ion peak area of *Z*-ligustilide was linear over the range of 24.4−487.7 μg/mL with slope of 1.16 × 10^5^. The coefficient of correlation (γ) was 0.9994. The limits of detection (LOD) and quantification (LOQ) for *Z*-ligustilide were 2.7 μg/mL and 3.9 μg/mL, respectively. The injection precision was determined by injecting successively standard for six times. The relative standard deviation (R.S.D.) was 1.6%, 2.3% and 3.5% at the concentration of 435.6 μg/mL, 123.8 μg/mL, and 34.2 μg/mL, respectively.

The short-term repeatability (six runs in 12 h at 4 °C) as well as the long-term repeatability (six runs in 24 h at 4 °C) of *Z*-ligustilide was calculated. The peak area of selected ion was relatively stable. The R.S.D.s of short- and long-term repeatability were 1.6−3.3% and 2.1−3.9% at the concentration of 435.6−34.2 μg/mL, respectively.

A comprehensive validation of the present method was conducted, a known amount of *Z*-ligustilide was added into samples of SWT, FSS, RA and RL, respectively, and extracted under the conditions mentioned above. Each extracted material was subjected to GC-MC, and the content of the analytes was calibrated. The recovery of the tested compound was 100.1%, 100.1%, 100.2% and 99.9% with relative standard deviation (R.S.D.) of 1.76%, 1.58%, 1.98% and 1.48% ( *n* = 5), respectively.

The contents of *Z*-ligustilide in SWT, FSS, RA and RL were determined by using the calibrated GC-MS. The compounds of the other peaks were identified by using GC-MS. The content of eight phthalides including 3-butylphthalide, *Z*-butylenephthalide, compound 6, senkyunolide A, *E*-butylenephthalide, neocnidilide, *Z* -ligustilide and *E*-ligustilide based on mass spectra in SWT, FSS, RA and RL, was determined or estimated by using *Z*-ligustilide as standard. The summary results are presented in Table 2, which shows the extractable content of the phthalides in FSS (2.0 g) was higher than total of that in RA (1.0 g) and RL (1.0 g), while the content of the phthalides in SWT (4.0 g) was lower than total of that in RA (1.0 g) and RL (1.0 g) because Radix Rehmanniae and Radix Paeonia possibly affected the extraction of the phthalides and furthermore, the extractable content of every phthalide in FSS and SWT is different than from single herbs. This is possibly a reason why there are some differences in their therapeutic action.

**Table 2:** Extractable content (mg) of eight phthalides based on mass spectra in SWT, FSS, RA and RL.

Compounds	SWT	FSS	RA	RL
3-Butylphthalide^a^	0.042 (0.8)^b^	0.086 (1.3)	0.007 (0.3)	0.096 (2.6)
*Z*-Butylenephthalide	0.116 (2.1)	0.194 (3.0)	0.030 (1.5)	0.160 (4.4)
Compound 6	0.022 (0.4)	0.046 (0.7)	+^c^	0.056 (1.5)
Senkyunolide A	0.166 (3.1)	0.278 (4.3)	0.002 (0.1)	0.444 (12.2)
*E*-Butylenephthalide	0.016 (0.3)	0.032 (0.5)	0.005 (0.2)	+
Neocnidilide	0.064 (1.2)	0.082 (1.3)	+	0.134 (3.7)
*Z*-Ligustilide	2.974 (54.8)	3.514 (54.6)	1.077 (53.0)	2.048 (56.3)
*E*-Ligustilide	0.070 (1.3)	0.100 (1.5)	0.027 (1.3)	0.048 (1.3)
Total	3.470 (63.9)	4.332 (67.3)	1.148 (56.5)	2.986 (82.1)

^a^ 3-Butylphthalide, *Z*-butylenephthalide, compound 6, senkyunolide A, *E*-butylenephthalide, neocnidilide and *E*-ligustilide were determined using *Z*-ligustilide as reference; ^b^ The data was presented as average of three replicates (R.S.D.< 2.5%). Injection volume 1 μL with split ratio of 40:1. Their extractable contents (mg) are relative to SWT (4.0 g), FSS (2.0 g), RA (1.0 g), and RL (1.0 g), respectively. The amount in parenthesis are the relative percentage content (%) of each phthalide in the different essential oils; ^c ^Under the limit of quantitation.

### 2.3. Inhibition of uterine contraction assay in vitro

Because phthalides have potent spasmolytic activity [22], and SWT, FSS, RA and RL are TCMs that have been used for centuries for treatment of women’s dysmenorrhea in China, the inhibitory activity of essential oils from SWT, FSS, RA and RL on uterine contraction was evaluated *in vitro*. The results (Table 3) showed that the inhibitory activity on mice uterine contractions *in vitro* (*P* < 0.05) of the essential oil were different at the same crude drug dosage (Table 3). 

**Table 3:** The effects of the investigated essential oils on uterine contraction *in vitro.*

Samples	Dosage(μg /mL)	Inhibiting ratio of frequency (%)	Inhibition ratio of contraction amplitude (%)	Inhibition ratio of muscle hypertonic (%)	ED_50_ (μg/mL)
control	-	8.78 ± 1.83	3.76 ± 0.52	13.25 ± 1.17	-
SWT	12.06	39.78 ± 1.64	13.10 ± 1.42	22.00 ± 2.33	12.34
FSS	12.55	51.67 ± 1.41	35.07 ± 2.56	48.10 ± 2.40	11.03
RA	12.87	27.18 ± 1.22	21.22 ± 1.12	20.21 ± 1.27	14.52
RL	6.82	24.98 ± 1.97	25.09 ± 1.92	23.45 ± 1.03	7.78
*Z*-Ligustilide	8.76	32.09 ± 3.08	32.28 ± 1.04	26.32 ± 1.28	5.60

Data are expressed as mean ± S.E.M. (*n* = 10).

**Figure 3:**
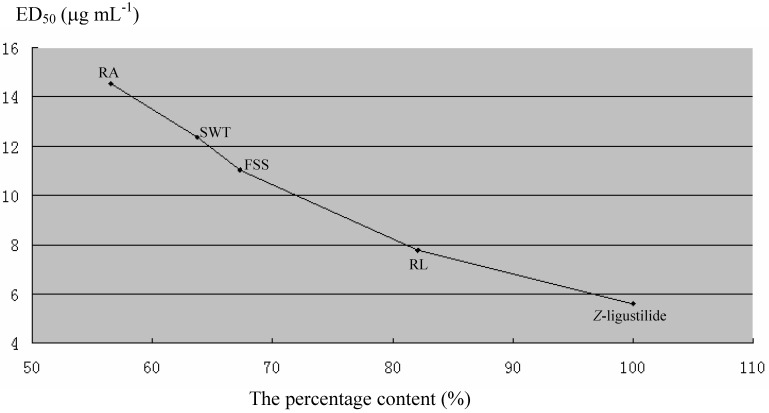
The percentage content (%) of total phthalides in essential oil－ effects on uterine contraction *in vitro*.

The ED_50_ of volatile oil from SWT, FSS, RA, RL, and *Z*-ligustilide, were 12.34, 11.03, 14.52, 7.78, and 5.60 μg/mL, respectively (Table 3). The activity of the essential oils is higher as the relative content of the phthalides in them increase (Figure 3). The results suggest that phthalides are the main bio-active constituents responsible for inhibiting mice uterine contraction *in vitro*.

## 3. Experimental

### 3.1. Plant materials

The crude Traditional Chinese Medicines Radix Angelica, Rhizoma Chuanxiong, Radix Rehmanniae and Radix Paeonia, were collected from Minxian (Gansu Province), Pengzhou (Sichuan Province), Huaiqing (Henan Province), and Bozhou (Anhui Province), respectively. All the crude herbs were identified by the corresponding author. Voucher specimens of all the samples (No. NJUTCM-20070611~20070614) were deposited with the Herbarium of Nanjing University of Chinese Medicine.

### 3.2. Standard preparation

(*Z*)-Ligustilide was separated and purified in our laboratory. The essential oils (20 mL) from RA were subjected to silica gel column chromatography eluting with *n*-hexane-ethyl acetate (100:1), followed by stepwise addition of ethyl acetate to yield eight fractions. Part of fraction 3 (1.0 mL) was dissolved with MeOH, and further purified by using preparative HPLC (RP_18_, 4 μm, 268 nm, MeOH-H_2_O, 70:30, Waters 2545-2767-UV2487) to give a yellow oily compound (0.5 mL) with a purity of more than 96.8% as tested by analytical HPLC. The structure was confirmed as (*Z*)-ligustilide by comparison of EI-MS (Table 1) and NMR data with reference [20]. 3-Butylphthalide, *Z*-butylene-phthalide and senkyunolide A were provided by Dr. Jieli Lü (Xinxiang Medical College, Henan Province, China). Ethyl acetate and petroleum ether for silica gel column chromatography were purchased from Nanjing Chemical Engineering Factory (Nanjing, China); methanol and acetone for HPLC and GC were purchased from TEDIA Company Inc. (USA). The deionized water was prepared from Millipore water purification system (Millipore, Milford, MA, USA) and was filtered with 0.45 μm membranes.

### 3.3. Sample preparation

Samples (100 g) of each herb containing RA and RL were crushed into small pieces. The mixed herbs of SWT, consisting of RA (100 g), RL (100 g), Radix Rehmanniae (100 g), and Radix Paeonia(100 g)*,* and another mixed herbs of FSS, consisting of RA (100 g) and RL (100 g) were processed by the same method. Essential oil samples were extracted by water distillation for 9 hours from the mixtures and each crude herb, respectively, using a set of standard apparatus, according to the procedure described in the *Pharmacopoeia of the Peoples’s Republic of China* [23]. The essential oils were isolated from water and dried over anhydrous sodium sulphate until the last traces of water were removed and then stored in the dark glass bottles at 4 °C. The essential oil yields of RA, RL, FSS, and SWT were 1.83%, 0.70%, 1.70%, and 0.81%, respectively. The essential oils were dissolved by acetone and filtered through 0.45 μm Econofilter (Agilent Technologies) before injection into the GC-MS system.

### 3.4. GC-MS analysis

The volatile components were analyzed using an Agilent 6890N gas chromatography system (Agilent Technologes, Palo Alto, CA, USA), equipped with a 5975B mass spectrometer and Agilent ChemStation software. A DB-5ms capillary column (0.25 mm × 30 m × 0.25 μm, dimethylpolysiloxane with 5% phenyl capping (Agilent Technologies) was used. The column initial temperature was kept at 80 °C, then temperature was increased from 80 °C to 100 °C at a rate of 15 °C/min and held for 3 min, and then to 250 °C at a rate of 3 °C and held for 5 min. Split injection was conducted with a split ratio of 40:1, injecting 1 μL of sample. The carrier gas was helium, at a flow rate of 1.0 mL/min. The spectrometer was operated in electron-impact (EI) mode and the ionization energy was 70 eV, the scan range was 35-550 amu and the rate was 3.71 s per scan. The ionization source temperature and accelerating voltage were 230 °C and 109 eV, respectively. The injector temperature was 250 °C.

### 3.5. Oxtocin-induced uterine contraction assay in vitro

Oxtocin induced mice uterine contraction was tested *in vitro* [30]. The experiments were performed in accordance with the Animal Ethics Committee of the Nanjing University of Chinese Medicine. In brief, non-pregnant sexually mature female Kunming strain mice (6-7 weeks, 18-22 g) were used in the experiments and the animals were provided free access to food and water. Rooms were in a cycle of 12 h of light (7:00-19:00) and 12 h of dark (from 19:00 to 7:00). Synchronistically estrus mice were pretreated with estradiol benzoate (s.c., 5 mg·kg^-1^) 24 hours prior to the study. On the third day, the mice were sacrificed by cervical dislocation and the uterus was isolated. The whole uterus was taken as one sample. The cervical end was tied to Perspex holder, and the two ovarian ends were put together as another end tied to an isometric force transducer. A resting tension of 1 g was applied for superfusion with oxygenated Krebs (95% O_2_, 5% CO_2_, pH = 7.3) at 37 °C. Equilibration period was not less than 45 min. Contractions were recorded by PowerLab/8s data recording system. (AD Instruments, Australia), Powerlab was linked to a Macintosh computer (Powermac 7200/120, Apple Inc, Cupertino, CA, USA) on which Chart software (v. 4.2.2 AD Instruments, Australia) was used to display and measure the tension changes in the tissue. The contraction frequency, amplitude and muscle hypertonic were used as the markers for uterine contraction. Inhibitions of drug on these three parameters are calculated as follows: Inhibition (%) = 100× (data before drug-data after drug)/data before drug.

## 4. Conclusions

Phthalides are a kind of important bio-active constituents found in SWT, FSS, RA and RL. In particular, ligustilide, butylenephthalide, and senkyunolide A have been widely investigated for their biological activities. In this paper, identification and comparative quantification by gas chromatography-mass spectrometry of the phthalides in essential oils from SWT, FSS and their constituting herbs including RA and RL, also containing abundant essential oils, were performed for the first time and the results showed that the extractable contents of each phthalide, including *Z*-ligustilide, *E*-ligustilide, *Z*-butylenephthalide, *etc*. varied after RA, RL were combined into a formulation, such as SWT and FSS. The changes of the essential oils from those herbs before and after preparation of the herbal medicines indicate that solubilization, chemical reactions and evaporation of some volatile components during decocting may induce changes in several components [24,25]. The comparative results were significant to help us to understand and use every herb and formula better and correctly. Furthermore, the inhibitory activity of the essential oils from SWT, FSS, RA and RL on uterine contraction *in vitro* was tested, and the results showed that the activity of the essential oil is higher as the content of the phthalides increases, which suggests that the phthalides are possibly the main active components inhibiting mice uterine contraction *in vitro*. All of the results suggested that comparative analysis of chemical components and pharmacological activities of each herb and formula is helpful in elucidating the active components in TCMs, and to reveal the compatibility mechanism of TCM formulae.
